# Low-temperature plasma improves the healing process of Achilles tendinopathy in a rat model

**DOI:** 10.3389/fbioe.2025.1656449

**Published:** 2025-11-26

**Authors:** Suying Wang, Juan Li, Qingyan Pan, Jiahe Li, Long Zhao, Yuhan Zhou, Gang Wang, Ming Zhou, Yangxiaoxue Liu, Aiguo Wang, Liping Huang, Xiaoran Chen

**Affiliations:** 1 Department of Comparative Medicine, Laboratory Animal Center, Dalian Medical University, Dalian, Liaoning, China; 2 Yantai Healing Technology Co. Ltd., Yantai, Shandong, China; 3 General Administration of Sports Qinhuangdao Training Base (China Football School), Qinhuangdao, China; 4 Shanxi Medical University, Taiyuan, Shanxi, China; 5 Department of Rehabilitation Medicine, The First Medical Center, Chinese PLA General Hospital, Beijing, China

**Keywords:** Achilles tendon, inflammation, collagen, blood flow, nitric oxide

## Abstract

**Background:**

Tendinopathy is a prevalent condition in orthopedics that significantly impairs tendon function. Recently, Low-temperature plasma (LTP) has emerged as an innovative treatment approach, showing promise in reducing inflammation and enhancing collagen synthesis during wound healing. This study aimed to evaluate the therapeutic benefits of LTP on Achilles tendinopathy.

**Methods:**

The effectiveness of LTP in treating Achilles tendinopathy was confirmed using an excessive exercise rat model, through assessments of biomechanical properties and pathological alterations. Type I and III collagen expression and mRNA levels of inflammatory factors were further detected. Blood flow and NO concentration finally examined.

**Result:**

LTP therapy markedly enhanced the biomechanical characteristics of the Achilles tendon, including maximum tension, stress, stiffness, and Young’s modulus. Correspondingly, histomorphometric analysis showed a significant improvement in the pathological alterations of the Achilles tendon and a decrease in the pathology score.Additionally, increased expression of collagen type I and decreased the ratio of collagen type III to collagen type I were observed after LTP treatment. Further, LTP treatment significantly inhibited the expression of cyclooxygenase-2 (COX-2),interleukin 1-β (IL-1β), tumor necrosis factor alpha (TNF-α), matrix metalloproteinase 1 (MMP-1), and matrix metalloproteinase 2 (MMP-2). Specifically, enhanced blood flow in the Achilles tendon was noted following LTP treatment. Intriguingly, LTP therapy also significantly increased nitric oxide (NO) levels in the skin, blood, and Achilles tendon.

**Conclusion:**

LTP exhibited a significant therapeutic impact on tendinopathy by inhibiting inflammation, promoting type I collagen synthesis, and enhancing blood flow, which may related to the active NO in LTP jet.

## 1 Introduction

Tendinopathy is a prevalent orthopedic disorder primarily caused by tendon overuse, with an adult incidence rate of 0.2%–0.3%—and athletes, as the highest-risk group, exhibit a morbidity rate of approximately 52% (van der Vlist et al., 2020; Kujala et al., 2005). Clinically, it presents with core symptoms including pain, joint dysfunction, and reduced exercise tolerance, which severely impair the quality of life and professional careers of affected individuals (Noriega-González et al., 2022). Biologically, the natural healing of injured tendons is slow and inefficient, largely due to the tissue’s inherent limitations: low regenerative capacity, sluggish cellular proliferation, and sparse vascular and nerve supply (Korntner et al., 2017). This poor self-repair ability, combined with the inadequacy of current treatments, exacerbates the clinical challenge of managing tendinopathy. Currently, the clinical standard for tendinopathy revolves around progressive loading exercises (e.g., eccentric strengthening protocols), which aim to stimulate tendon remodeling. However, these approaches often deliver inconsistent therapeutic effects and require a prolonged duration to take effect (Goncalves et al., 2025; Morya et al., 2024). Pharmacologically, non-steroidal anti-inflammatory drugs (NSAIDs) are occasionally used for short-term pain relief, but long-term use is discouraged—they do not improve tendon structural integrity, and even after intervention, tendon function rarely reverts to its pre-injury state (Maffulli et al., 2010; Jiao et al., 2023). Given the limitations of existing therapies, there is an urgent need to develop novel, effective treatment strategies for tendinopathy.

Tendon healing encompasses three distinct yet interrelated stages. Initially, at the injury site, macrophages and other inflammatory cells secrete various inflammatory mediators during the early inflammation phase (Baugé et al., 2015). Prolonged or excessive inflammation leads to tendon cell death, matrix degradation, and the formation of peritendinous scars, which can effectively impede tendon structural and strength recovery and excursion (Shen and Lane, 2023). In addition, key pro-inflammatory cytokines (e.g., TNF-α, IL-1β) are significantly upregulated in degenerated tendon tissues. These cytokines promote the recruitment of inflammatory cells to the lesion site, exacerbating local inflammatory infiltration; they also upregulate the expression of matrix metalloproteinases (MMPs), accelerating the degradation of extracellular matrix (ECM) components such as type I collagen (Kessler et al., 2014; Shakibaei et al., 2011; Huang et al., 2025). Meanwhile, COX-2 mediates the production of prostaglandin E2 (PGE2)—the major prostaglandin (PG) subtype closely linked to pain and inflammation in tendinopathy (Choi et al., 2020). The subsequent stage is characterized by the synthesis of a large amount of collagen III by tendon cells, serving as a temporary repair mechanism. The final phase involves remodeling, where type I collagen, a subtype more conducive to long-term tendon functional recovery, gradually replaces type III collagen. Higher levels of type I collagen in the tissue correlate with improved restoration of tendon stiffness, tensile strength, and resistance to re-injury (He et al., 2022). In addition, effectively increased blood circulation is also an essential factor for tendon recovery (Kubo, 2016). Therefore, treatment strategies that focus on reducing inflammation, promoting collagen transformation, and improving blood flow are fundamental to the clinical management of tendon injuries (Jiao et al., 2023; Oshita et al., 2016).

Plasma medicine is an emerging interdisciplinary field that has been applied in bacterial inactivation, wound healing, cancer therapy, etc (Choi et al., 2021). Low-temperature plasma (LTP), a versatile technology in plasma medicine, employs devices that generate reactive oxygen/nitrogen species (RONS). These RONS are recognized for their primary therapeutic effects on cells and tissues by targeting the intrinsic mechanisms within eukaryotic cells (Hirst et al., 2016; Tanaka et al., 2021). Specially, LTP has been demonstrated to regulate inflammatory responses, encourage collagen synthesis, and stimulate angiogenesis during treatment processes (Ge et al., 2022; Kim et al., 2022). However, the clinical application of low-power LTP devices has largely been confined to treating superficial biological tissues, such as in wound healing, atopic dermatitis, and psoriasis (Choi et al., 2021; Kim et al., 2022; Choi et al., 2017). By contrast, our newly developed high-power LTP (hLTP) device offers key advantages: its effective treatment area is significantly expanded (from the conventional 1–5 cm^2^ to >20 cm^2^), it uses air instead of rare gases as the gas source, and the concentration and diversity of its reactive species are substantially enhanced. Beyond superficial use, this device has demonstrated marked efficacy in promoting wound healing (Guo et al., 2022a), and offering potential treatments for organ diseases like cisplatin-induced nephrotoxicity (Guo et al., 2022b). These observations suggest that the novel high-power LTP device can therapy deeper tissue and organ injuries. However, whether LTP can effectively treat tendinopathy has not been determined yet.

In the present study, we validated the therapeutic efficacy of high-power LTP on tendinopathy through an overuse Achilles tendinopathy rat model, examining its anti-inflammatory, collagen-conversion, and blood flow enhancement capabilities. This study reveals for the first time the therapeutic effect of high-power LTP on tendinopathy, offering significant insights into its potential applications for deep tissue and organ diseases.

## 2 Materials and methods

### 2.1 Study design

All experimental protocols were approved by the Animal Care and Use Committee of Dalian Medical University, and experiments were performed at the Laboratory Animal Center of Dalian Medical University. Sprague-Dawley rats, aged 7 weeks and weighing between 250 and 300 g, were procured from Liaoning Changsheng Biotechnology Co. Ltd (Liaoning Province, China). Prior to the commencement of the experiments, the rats were allowed a 7-day acclimatization period during which they were fed and housed under standard conditions: laboratory temperature 22 °C ± 2 °C, humidity 60% ± 5%, 12-h day/night cycle and quiet environment. Throughout the study, all animals had unrestricted access to food and water. Specific treatments were administered in accordance with the experimental protocols outlined below.

**FIGURE 1:**
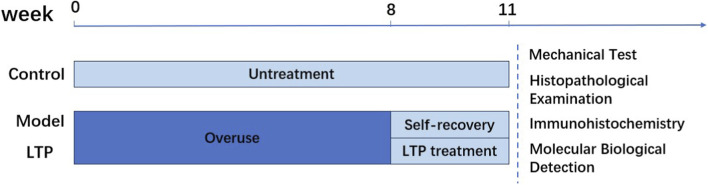
Schedule of the experimental protocol.

The design of the primary study is shown in Figure 1. Animals were randomly assigned to three groups using GraphPad Prism 9.0, with allocation concealment achieved via sealed envelopes. The healthy control group was maintained under normal conditions. Conversely, both the model group and the LTP treatment group were subjected to 8 weeks of overuse, resulting in damage to the Achilles tendon. Following the modeling process, the model group underwent a self-recovery period of 3 weeks, whereas the LTP group received LTP treatment for the same duration. Subsequently, after a total of 11 weeks, all animals were euthanized in a sealed chamber by exposure to 100% carbon dioxide at a flow rate of 20% chamber volume per minute (5 L/min for a 25 L chamber). The chamber was pre-filled with CO_2_ for 30 s before introducing the animals, and euthanasia was confirmed by the absence of respiratory movements and righting reflex for 5 min. Following euthanasia, Achilles tendon tissues were collected for additional biological analyses. Samples underwent random processing, with assay-conducting laboratory staff blinded to group assignments to minimize subjective bias. This method complied with the animal care protocol approved by the Institutional Animal Care and Use Committee (Ethics Approval No. AEE22033).

### 2.2 Achilles tendinopathy model preparation

Rats were trained for 2 weeks with daily increments in distance (25–1,065 m/day), speed (5–13.4 m/min), and incline (0°–10°) following a 1-week acclimatization period. Subsequently, the rats were trained for 6 weeks to run at a consistent 10°incline. Each day, the rats began with a 10-min warm-up run at an accelerated pace, followed by a 10-min cool-down run at a slower pace. Throughout this training regimen, they operated at their maximum speed of 18 m per minute for a total duration of 60 min (Jafari et al., 2015).

### 2.3 hLTP treatment

**FIGURE 2:**
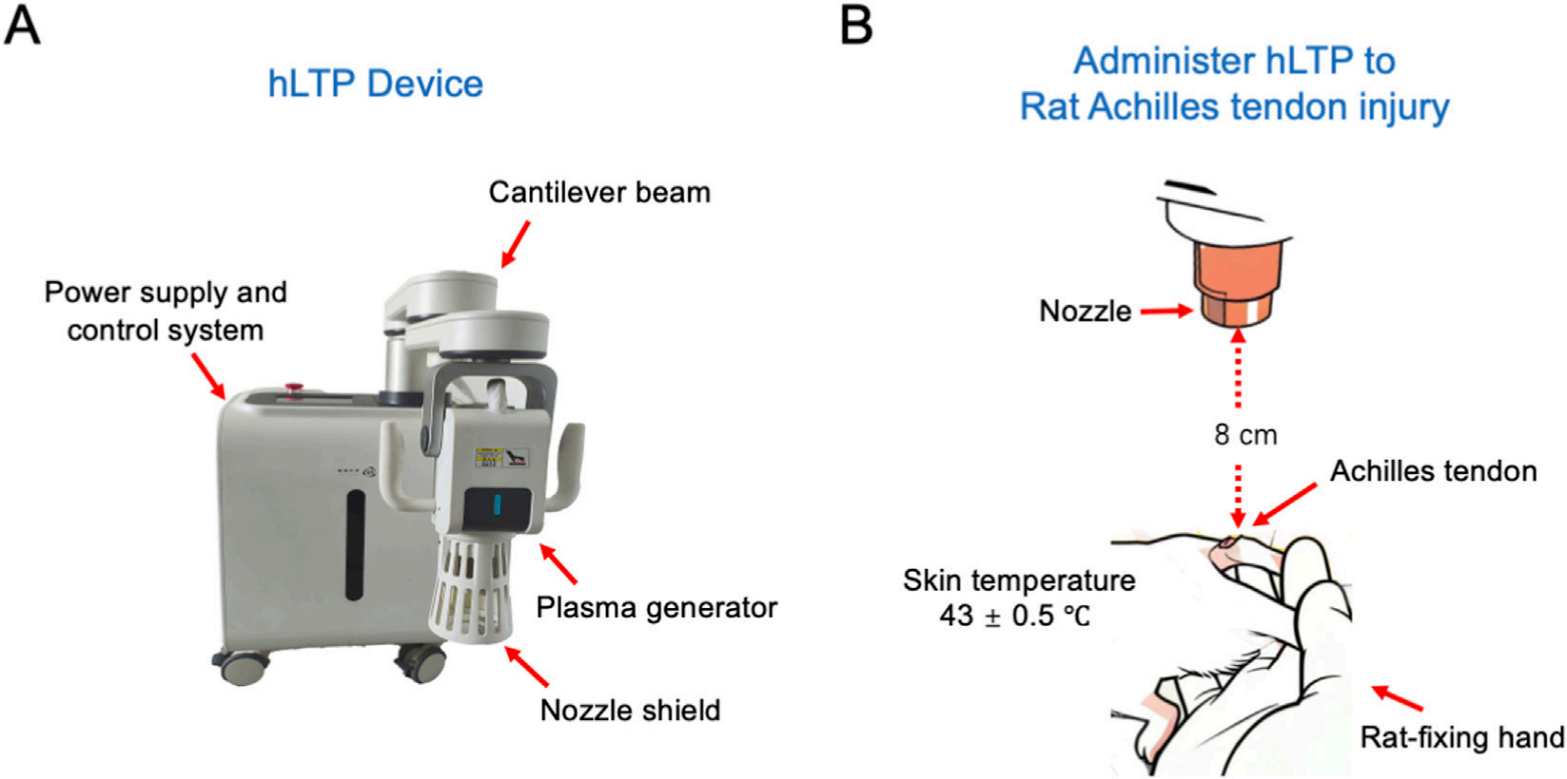
hLTP device and rat Achilles tendon treatment protocol. **(A)** Schematic diagram of the hLTPdevice, illustrating its core components. **(B)** Schematic diagram showing the application of hLTP treatment for rat Achilles tendon injury.

Rats in the hLTP treatment group received bilateral Achilles tendon treatment using a novel hLTP device (Yantai Hailing Biotechnology Co., Ltd., Registration No. QH20200272; Figure 2A), with treatment parameters strictly controlled: temperature maintained at 43 °C ± 0.5 °C, the plasma jet nozzle positioned 8 cm away from the target tendon tissue (Guo et al., 2022a), and each treatment session (twice daily) involving 3 min of plasma jet exposure to the Achilles tendon; during treatment, rats remained awake throughout the procedure, and laboratory personnel manually restrained the rats to stabilize their posture, ensuring the Achilles tendon regions were optimally exposed for targeted treatment of each tendon individually (Figure 2B).

### 2.4 Tendon mechanical testing

Immediately after euthanasia, rat Achilles tendon tissues were stored at −80 °C for subsequent mechanical testing. Prior to testing, the width and thickness of each tendon were measured with a vernier caliper (accuracy: 0.01 mm), and the cross-sectional area was calculated using the formula: Cross-sectional area = π × (major axis/2) × (minor axis/2). For specimen gripping, a custom-machined clamping device was used: one end firmly secured the calcaneus (to avoid damage to the tendon-bone junction), and the other end anchored the tendon-muscle junction, ensuring no relative displacement during testing. After clamping, specimens were mounted on a universal mechanical testing system (Instron Model 5982) for uniaxial tensile testing. The test protocol was as follows: a constant displacement rate of 5 mm/min was applied until the tendon completely failed, and key mechanical parameters (ultimate failure load, maximum tensile stress, stiffness, Young’s modulus) were recorded in real time via the system’s software. Throughout the entire testing process, the tendon specimen was continuously moistened with normal saline to prevent desiccation-induced changes in its mechanical properties.

### 2.5 Hematoxylin and eosin (H&E) staining

The Achilles tendon tissues were fixed in paraffin and then longitudinally sectioned using a microtome to produce consecutive sections, each with a thickness of 5 μm. These sections were subsequently stained with hematoxylin and eosin. For evaluation, Five non-overlapping fields of view (FOVs) were selected per section, with each FOV measuring 1,000 × 1,000 μm (at ×200 microscopic magnification). FOVs were specifically chosen from the mid-portion of the tendon lesion area (the core region of tendinopathy, which is the primary site of pathological changes and therapeutic response) to ensure consistency across samples. Both FOV selection and subsequent histological assessment were performed by a researcher blinded to the experimental groups to avoid subjective bias. Bonar’s histological grading scale was utilized to assess the samples, which encompasses criteria such as collagen bundle characteristics, neovascularization, tendon cell morphology, and the presence of ground substance. Higher scores on this scale indicate poorer tendon healing.

### 2.6 Immunohistochemical staining

Immunohistochemical techniques were utilized to analyze the distribution of type I collagen and type III collagen within the Achilles tendon. The methodology involved dewaxing paraffin sections to eliminate water, antigen retrieval through boiling, blocking with 3% bovine serum albumin (BSA), and subsequent overnight incubation with anti-rabbit type I or III collagen polyclonal antibodies (Servicebio, China) at a dilution of 1:800. Following this, biotinylated goat anti-rabbit antibody was applied as a secondary antibody for 1 h. Color development was achieved using 3,3-diaminobenzidine tetrahydrochloride (DAB), followed by restaining with hematoxylin. Five non-overlapping, well-stained FOVs were selected per section, with each FOV sized 1,000 × 1,000 μm (at ×200 microscopic magnification). FOVs were sampled from the tendon lesion area (consistent with the H&E staining analysis region) to ensure alignment with the pathological focus. Both FOV selection and quantitative analysis (performed using ImageJ software to measure the integrated optical density [IOD] of collagen-positive signals) were conducted by a researcher blinded to the experimental groups to ensure result objectivity.

### 2.7 RT-qPCR

For Achilles tendon tissue grinding, given the relatively hard texture of tendon tissue that makes conventional homogenizers ineffective for disruption, we first placed the tissue sample into a pre-chilled mortar and added sufficient liquid nitrogen to submerge the tissue. Once the tissue was fully frozen, we used a pestle to grind it rapidly into a powder; during this process, additional liquid nitrogen was continuously replenished to keep the tissue in a frozen state (to prevent RNA degradation). After the tissue was ground into a uniform powder, it was immediately transferred to a centrifuge tube containing Trizol reagent and gently mixed to initiate RNA extraction.

Total RNA was then extracted strictly following the manufacturer’s instructions provided by the Trizol Reagent (Saiwen Innovation (Beijing) Biotechnology Co., Ltd.).Subsequently, RNA was reverse transcribed into complementary DNA (cDNA) using a transcription RT kit (TransGen Biotech, China). The qPCR procedure was conducted using the SYBR Green fluorometric method.

The target genes analyzed in this study included inflammatory factors (*TNF-α, IL-1β, COX-2*), matrix metalloproteinases (*MMP-1, MMP-2*), and the housekeeping gene *GAPDH* (used as the internal reference gene for data normalization). The specific primer sequences for these genes are listed in Table 1.

**TABLE 1:** RT-qPCR primer sequences.

Genes	Forward (5′to3′)	Reverse (5′to3′)
*TNF-α*	CCA​GGT​TCT​CTT​CAA​GGG​ACA	AAG​GGC​TCT​TGA​TGG​CAG​AG
*IL-1β*	AAC​AGC​AAT​GGT​CGG​GAC​AT	ACT​GCC​CAT​TCT​CGA​CAA​GG
*COX-2*	CCC​ATG​GGT​GTG​AAA​GGA​AA	GGG​ATC​CGG​GAT​GAA​CTC​TC
*MMP-1*	CAT​ACT​GTA​CTG​AGA​GGA​TTC​CCC​ACA​GA	ACA​TCA​TCA​ACT​TTA​TCG​TCA​ATT​CCA​GG
*MMP-2*	ACA​CCT​GAC​CTG​GAC​CCT​GA	TTC​CCC​ATC​ATG​GAT​TCG​AG
*GAPDH*	ACT​TTG​GCA​TCG​TGG​AAG​GG	AGG​GAT​GAT​GTT​CTG​GGC​TG

### 2.8 Optical emission spectroscopy (OES) measurements

Elemental identification and quantification in LTP were performed using an Avaspec-ULS2048 optical emission spectrometer (Avantes) with a spectral resolution of 0.5 nm. For measurements, the optical fiber probe was positioned perpendicularly 8 cm below the instrument nozzle, with carrier gas flow maintained at 10 m^3^/h. Incident light from a 5 mm-diameter spot was collimated via a 10 mm focal length lens. All spectral data were acquired and analyzed quantitatively using AvaSoft 7.8 spectroscopy software.

### 2.9 NO content assay

After 2 weeks of LTP treatment (to stabilize the physiological effects of LTP), NO content was measured immediately following the final 3-min LTP session on rat Achilles tendons. This NO assay was designed as an independent experiment, using only healthy rats that had not undergone treadmill training. For sample collection: Achilles tendon skin, the Achilles tendon itself, and blood samples were harvested from experimental rats (all healthy, non-treadmill-trained animals). NO content determination: A total nitric oxide assay kit (Beyotime, Shanghai, China) was used per the manufacturer’s instructions. Briefly, samples were processed following the kit protocol; absorbance at 540 nm was measured with a microplate reader (BIO-RAD, CA, United States), and NO content was calculated using a parallel-generated standard curve.

### 2.10 Hot wind treatment

To isolate the effect of thermal stimulation (a potential confounder) from the NO-mediated effects of LTP in blood flow detection, a dedicated hot air experimental group was established, with procedures as follows: A constant-temperature hot air device delivered continuous hot air to the Achilles tendon region of resting rats. Key parameters were standardized to match the LTP treatment group for consistency: hot air temperature was set to 43 °C ± 0.5 °C (same as the LTP device’s thermal output), the air outlet was fixed 8 cm from the tendon tissue surface (consistent with the LTP nozzle-to-tissue distance), and intervention duration was 3 min (identical to LTP treatment time). All environmental conditions (e.g., humidity, lighting) during hot air treatment were kept consistent with the LTP group to eliminate external variables. This group was specifically designed for comparative analysis of blood flow responses, enabling differentiation between thermal-induced and NO-induced changes in tendon perfusion.

### 2.11 Blood flow assay

The blood flow in rat Achilles tendons was measured immediately after a 3-min low-temperature plasma (LTP) or hot wind treatment. This assay was designed to test the immediate effects of these interventions and conducted as an independent experiment, using only healthy rats that had not undergone treadmill training. The rats were maintained under continuous anesthesia using isoflurane, and the surface skin covering the Achilles tendon was incised using an electric knife to ensure there was no bleeding during the procedure. This exposed the intact Achilles tendon. A laser emitted from a laser diffusion flow imager (RFLSI ZW, Shenzhen, China) was directed onto the exposed Achilles tendon. Subsequently, an appropriately sized region of interest (ROI) area was delineated, and a signal was recorded every 5 s for the measurement of Achilles tendon perfusion. Stable data collected over a period of 10–20 min was utilized for statistical analysis.

### 2.12 Statistical analysis

Experimental data were analyzed using GraphPad Prism 8 software and presented as mean ± SEM (standard error of the mean). For pairwise comparisons between two groups, two-tailed unpaired Student’s t-tests were performed to assess statistical differences.For multiple-group comparisons, the following steps were implemented: first, data normality was tested using the Shapiro-Wilk test. If data conformed to a normal distribution, one-way analysis of variance (ANOVA) was conducted; when ANOVA results indicated a statistically significant overall difference (p < 0.05), Tukey’s honest significant difference (HSD) test was further applied for *post hoc* multiple comparisons (this step specifically addresses the correction for multiple testing to avoid type I errors). If data did not meet the normality assumption, the non-parametric Kruskal–Wallis H test was used to compare differences across multiple groups.

## 3 Results

**FIGURE 3:**
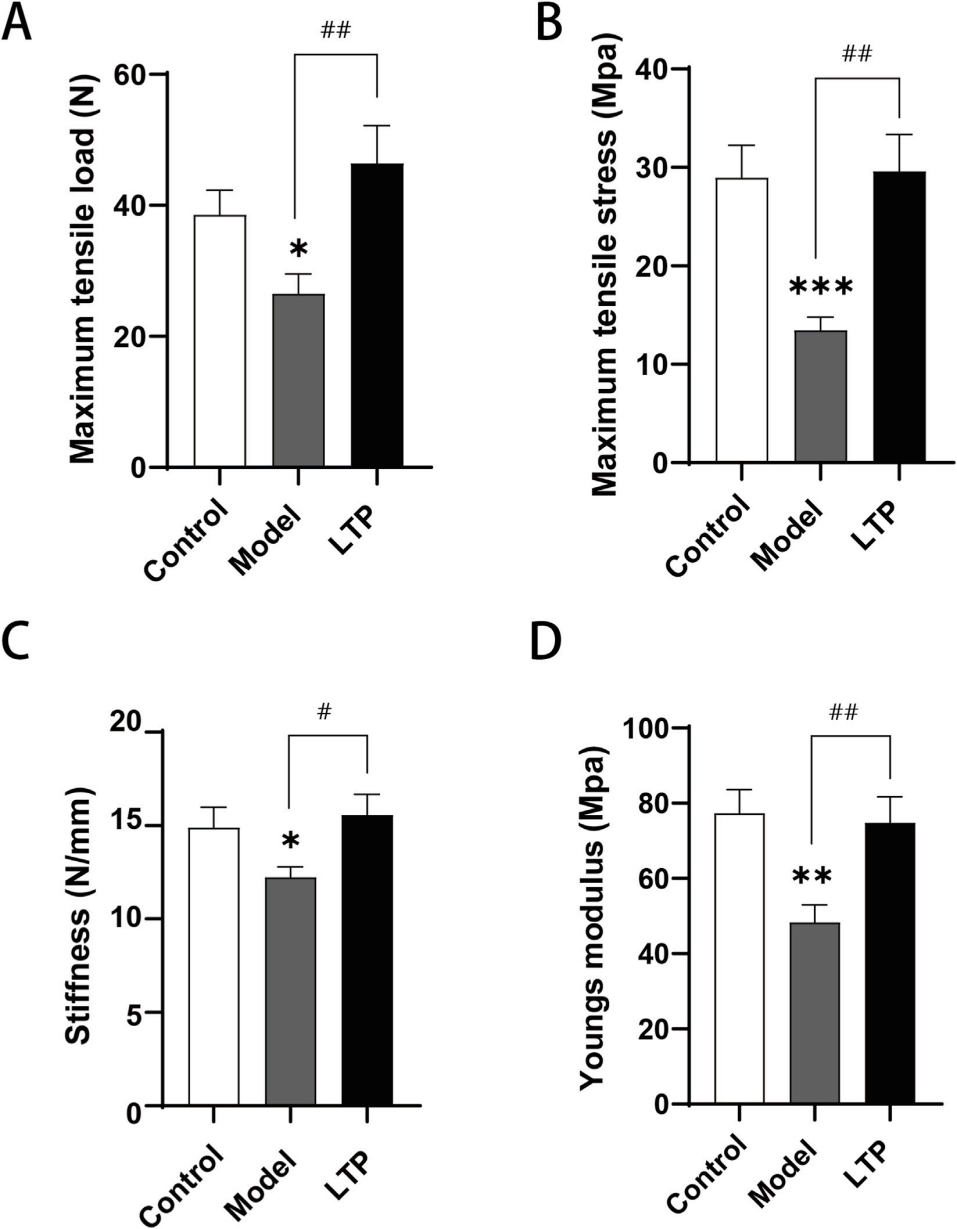
Biomechanical analysis of Achilles tendon tissues. Achilles tendon biomechanical properties were measured using a universal testing machine (Instron Model 5982), with the following parameters quantified: **(A)** maximum tensile load, **(B)** maximum tensile stress, **(C)** stiffness, and **(D)** Young’s modulus. n = 6–8. Statistical significance: *P < 0.05, **P < 0.01, ***P < 0.001 compared with the Control group; #P < 0.05, ##P < 0.01 compared with the Model group.

**FIGURE 4:**
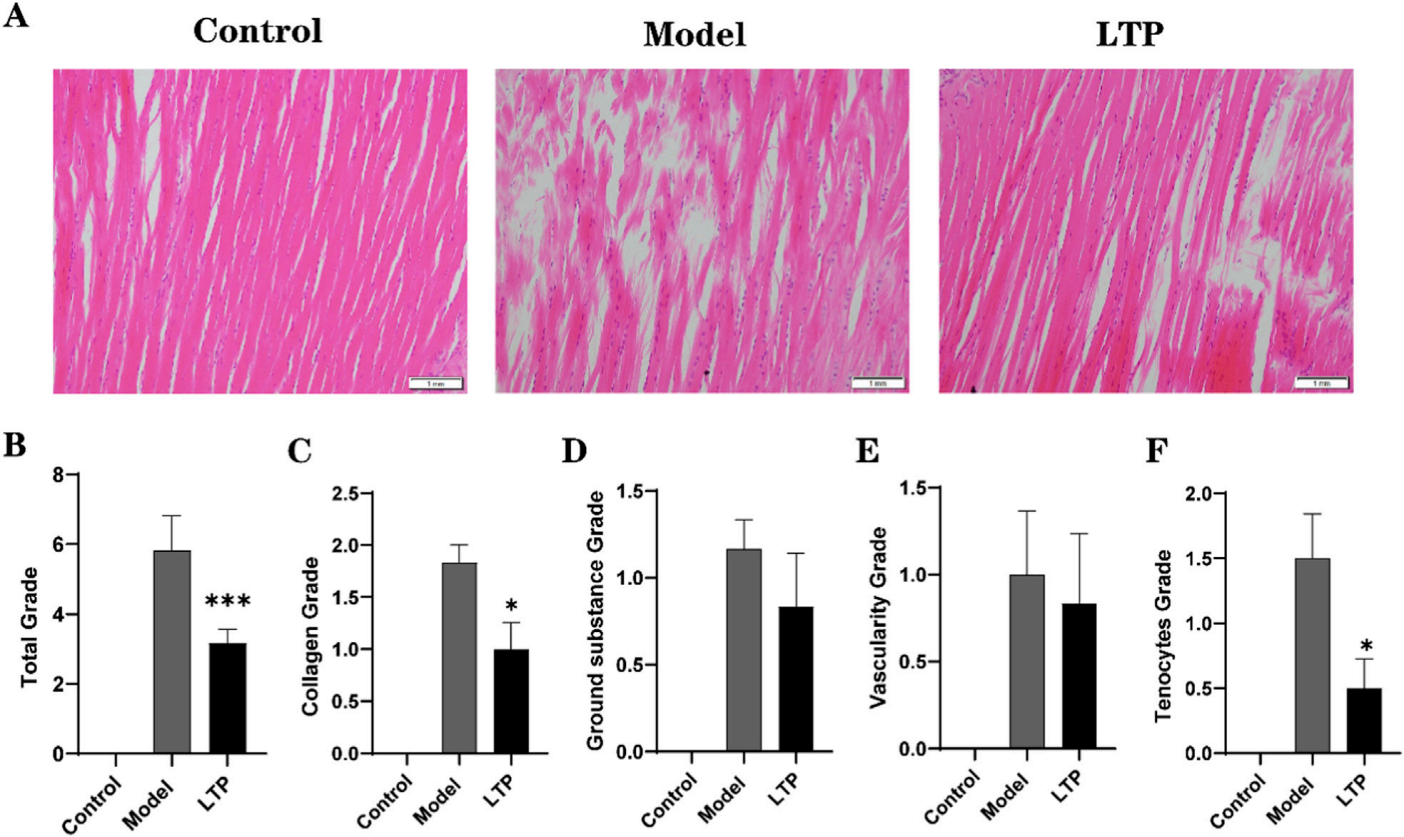
Bonar scoring of tendon tissues was performed based on the histopathological examination. **(A)** HE staining. **(B)** Total Grade. **(C)** Collagen Grade. **(D)** Ground substance Grade. **(E)** Vascularity Grade. **(F)** Tenocytes Grade. n = 6. Statistical significance: *P < 0.05, ***P < 0.001 compared with the Model group.

**FIGURE 5:**
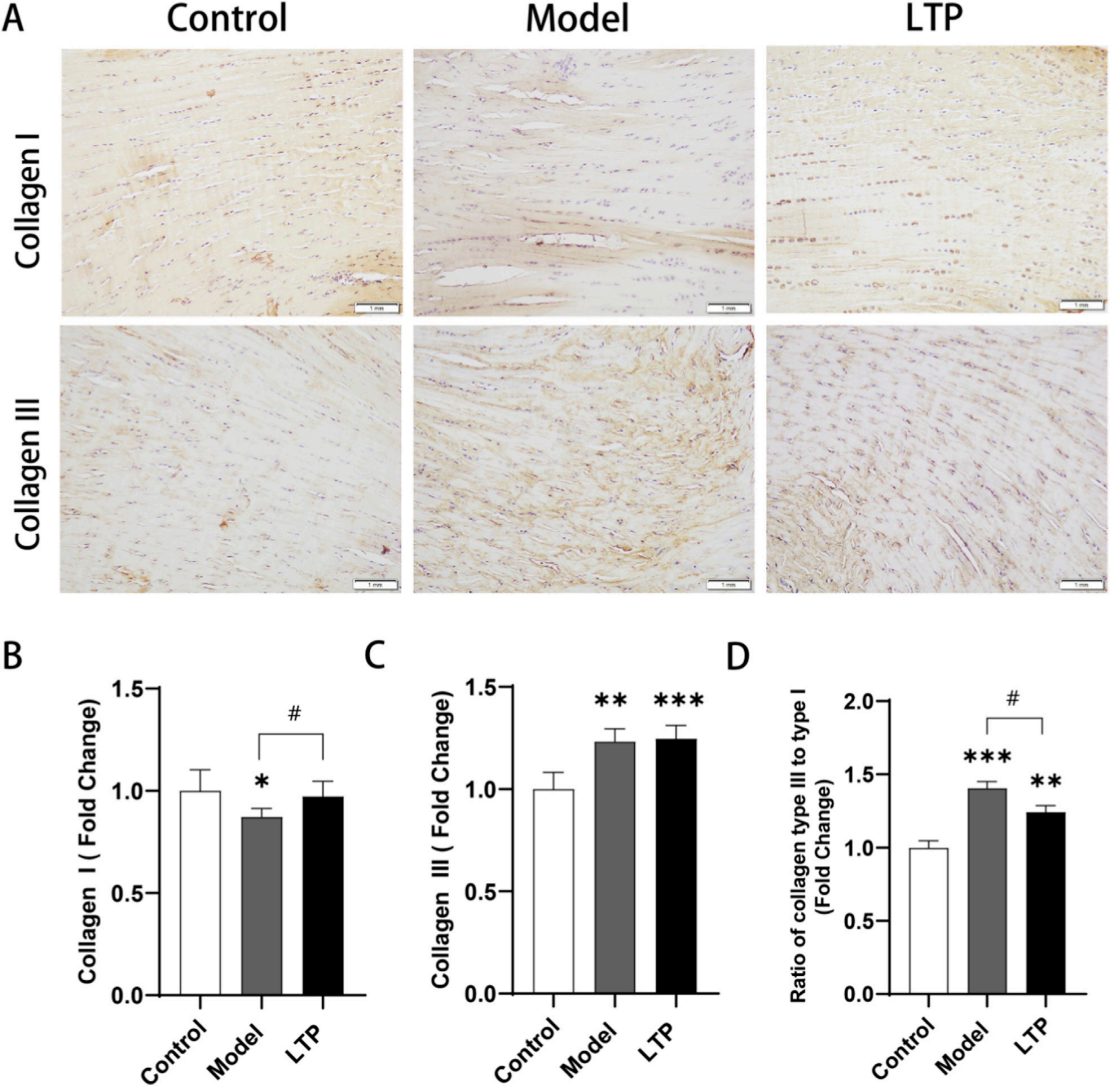
Immunohistochemical staining for Collagen Ⅰ and Collagen Ⅲ. **(A)** Type I and III Collagen staining. **(B)** Relative quantification of type ICollagen. **(C)** Relative quantification of type ⅢCollagen. **(D)** Ratio of collagen type III to type I. n = 5. Statistical significance: *P < 0.05, **P < 0.01, ***P < 0.001 compared with the Control group; #P < 0.05compared with the Model group.

**FIGURE 6:**
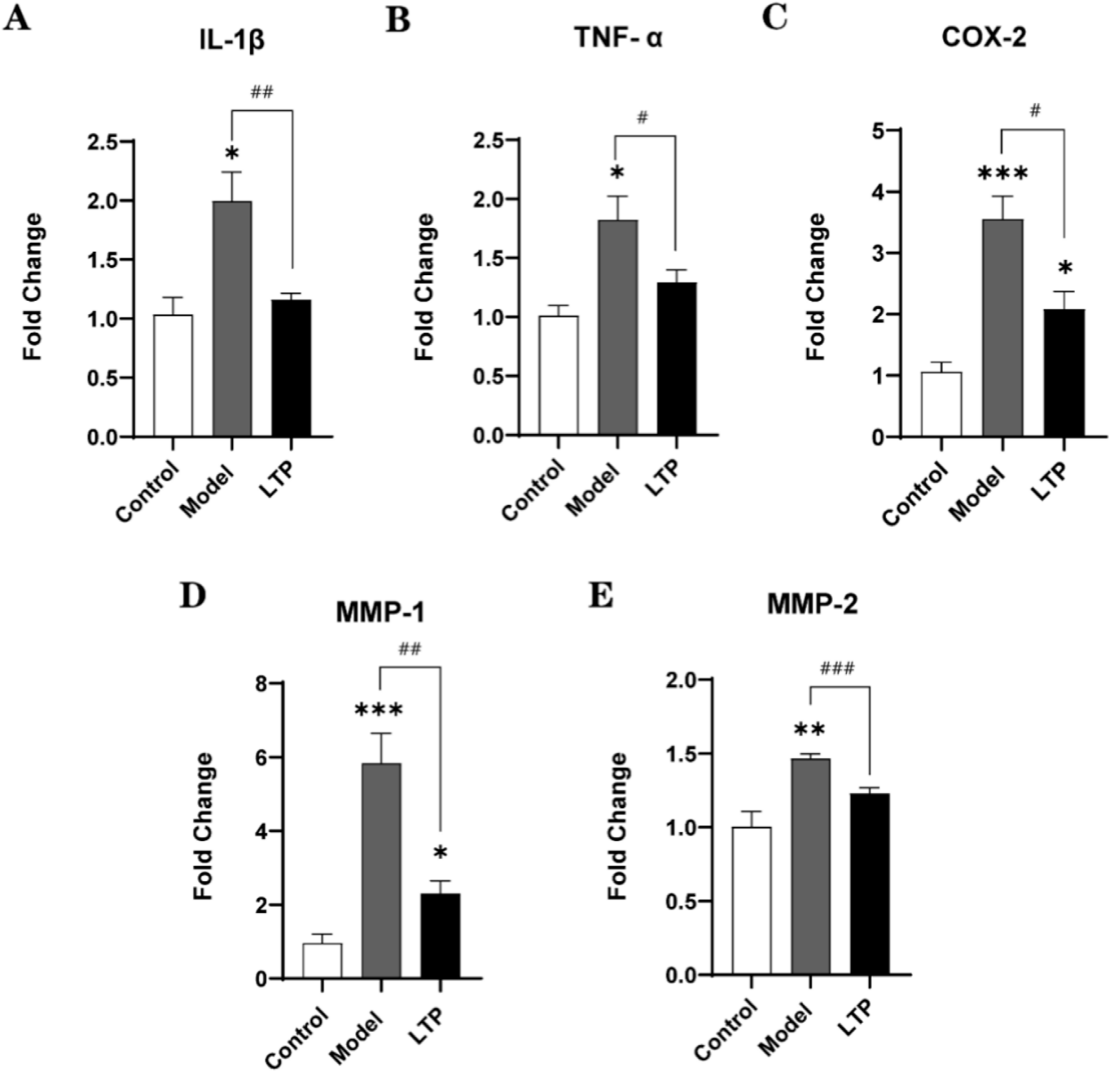
The mRNA levels of various inflammatory factors as analyzed by real-time RT-qPCR assay. **(A)** IL-1β. **(B)** TNF-α. **(C)** COX-2. **(D)** MMP-1. **(E)** MMP-2. n = 6. Statistical significance: *P < 0.05, **P < 0.01, ***P < 0.001 compared with the Control group; #P < 0.05, ##P < 0.01, ###P < 0.001 compared with the Model group.

All experiments utilized a high-power low-temperature plasma (hLTP) device with the following parameters: working gas (air), output power (1000 W), treatment distance (8 cm), and pulse frequency (60 kHz); each Achilles tendon received 3-min treatments, twice daily, over a 3-week period. The animal study supporting Figures 1–6 included 7-week-old male Sprague-Dawley rats (250–300 g) randomized into three groups: Healthy Controls (no tendinopathy induction or LTP), Tendinopathy Models (tendinopathy induced via 8 weeks of treadmill overtraining without LTP), and LTP-treated animals (tendinopathy induced as above, followed by 3 weeks of LTP treatment as described). Achilles tendon tissues were collected immediately after the final LTP session for analyses presented in Figures 1–6.

### 3.1 Biomechanical test for achilles tendon tissues

The biomechanical test was conducted after 21 days of LTP treatment. A comparison between the LTP group and the model group revealed significantly higher maximum tensile load in the LTP group (Figure 3A). Similarly, Figures 3B,C, Cillustrate a substantial increase in stiffness and maximum tensile stress in the LTP group compared to the model group.Furthermore, treatment with LTP resulted in improved material-level properties of the tendon tissue. This was evident from the considerably higher Young’s modulus observed in the LTP group compared to the model group (Figure 3D).

### 3.2 Histological evaluation of achilles tendon tissues

To evaluate the therapeutic effect of LTP on Achilles tendon injuries, we conducted HE staining and Bonar scoring on tendon tissues (Figure 4). In comparison to the healthy control group, the model group exhibited disrupted collagen fiber structure, disorganized arrangement, thickened or severely interrupted fibers, and a higher number of fibroblasts with larger cytosol. However, significant improvements in collagen fiber structure and cell morphology were observed in the LTP group compared to the model group (Figure 4A). These observations were further supported by the Bonar score, which indicated that the LTP group had a significantly lower total score than the model group (Figure 4B). In terms of Collagen Grade and Tenocytes Grade scoring, the LTP group was significantly lower than that of the model group (Figures 4C,D). Although no significant differences were observed in Ground Substance Grade and Vascularity Grade scoring between the two groups, there was a trend towards a decrease in the LTP group (Figures 4E,F). These findings provide evidence that LTP treatment can significantly improve Achilles tendon injury in rats.

### 3.3 Immunohistochemical staining oftypeIand type Ⅲ collagen in achilles tendon tissues

Immunohistochemical staining of type I collagen and type III collagen was performed to evaluate the restorative effects of LTP. In the healthy control group, type I collagen was prominently expressed and neatly arranged, while type III collagen was less expressed, allowing for clear visualization of the nuclei of tendon cells. Conversely, in the model group, type III collagen expression was significantly increased while type I collagen expression was significantly decreased compared to the control group (Figures 5A–C). Although there was no statistically significant difference in type III collagen expression between the LTP group and the model group, type I collagen expression was notably higher in the LTP group (Figures 5A–C). Additionally, the ratio of type III collagen to type I collagen was consistently lower in the LTP group compared to the model group (Figure 5D). These findings suggest that LTP treatment can improve Achilles tendon injury by promoting the conversion of type III collagen to type I collagen.

### 3.4 Analysis of various inflammatory factors in achilles tendon tissues

To assess how hLTP treatment regulates inflammatory responses in the Achilles tendinopathy model, we measured the mRNA expression levels of inflammatory cytokines and related molecules in tendon tissues using real-time RT-qPCR.As shown in Figures 6A–E, the mRNA levels of the examined inflammatory factors and matrix metalloproteinases (MMPs) were significantly higher in the tendinopathy model group than in the healthy control group. In contrast, compared with the model group, LTP treatment led to a marked reduction in the mRNA expression of three key pro-inflammatory factors (IL-β, TNF-α, COX-2) and two MMPs (MMP-1, MMP-2) (Figures 6A–E). These results demonstrate that LTP treatment alleviates Achilles tendon injury, at least in part, by suppressing the local inflammatory response.

### 3.5 Nitric oxide and blood flow detection in achilles tendon tissues

**FIGURE 7:**
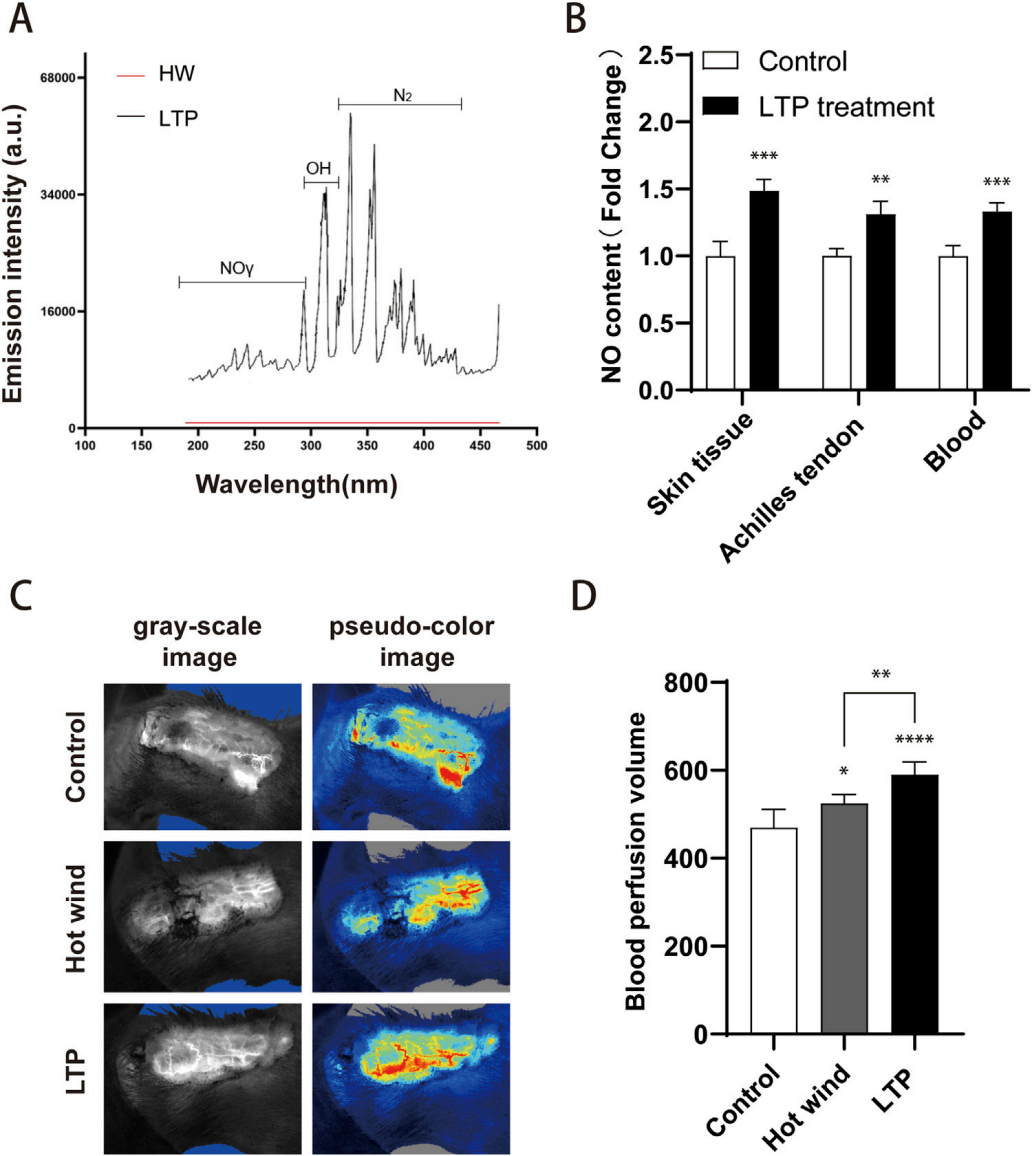
Detection of nitric oxide (NO) and blood flow in Achilles tendon tissues. **(A)** Analysis of plasma emission spectra; **(B)** Measurement of NO content: NO concentrations in skin, tendon tissue, and blood were quantified using the Griess reagent method (Beyotime, Shanghai, China); **(C)** Representative grayscale and pseudo-color images of Achilles tendon blood flow via a laser diffusion flow imager (RFLSI ZW, Shenzhen, China); **(D)** Evaluation of Achilles tendon blood perfusion. n = 4–7. Statistical significance: *P < 0.05, **P < 0.01, ***P < 0.001, ****P < 0.0001 compared with the Control group; ##P < 0.01, ###P < 0.001 compared with the Hot wind group.

OES analysis revealed strong characteristic γ-band NO signals, while hot air controls (matched to the plasma treatment temperature) exhibited negligible NO signals (Figure 7A). These results indicate that NO, a major chemically active substance, is detected exclusively in the novel hLTP device. To assess the penetration of NO into deep tissues, nitrate and nitrite levels were measured. The results revealed a significant increase in nitrite concentration in the skin, Achilles tendon tissue, and blood after 3 min of LTP treatment compared to the control group (Figure 7B). To investigate whether NO from LTP could enhance blood circulation, the blood perfusion within the Achilles tendon was measured using laser scatterometry. Hot air (matched to the plasma treatment temperature) was used as a control to exclude the effect of pure thermal stimulation on blood perfusion, allowing specific evaluation of the biological effects of NO. Both hot air and LTP treatments notably increased blood perfusion compared to the untreated control group. However, the blood perfusion in the LTP group was significantly higher compared to the hot air group (Figures 7C,D). This finding indicates that the bioactive NO generated by hLTP likely plays a key regulatory role in mediating tendinopathy treatment—providing critical experimental evidence and a clear direction for further dissecting hLTP’s therapeutic mechanism and optimizing its treatment protocol.

## 4 Discussion

The use of current Low-power LTP devices is restricted to superficial applications such as bacterial sterilization, wound healing, and skin cancer treatment due to their limitations, including a small effective treatment area (1–5 cm^2)^, low gas flow rate (0.12–0.3 m^3^/h), short working distance (8–20 mm), and the use of rare gas sources. To overcome these constraints and extend the clinical applicability of LTP technology, we have developed a novel high-power LTP equipment with a free gas supply (air), large effective treatment area (>20 cm^2^), high gas flow rate (10 m^3^/h) and long working distance (8 cm). In addition, because of the high power, it can generate a high concentration of active substances with strong penetration capabilities, enabling it to reach and treat deep tissues and organs (Guo et al., 2022a). In preclinical trials, this technology has been demonstrated not only to facilitate skin wound healing but also to offer protection to deep tissue from renal damage following cisplatin chemotherapy (Guo et al., 2022a; Guo et al., 2022b). In our current study, we provide the first evidence of LTP’s effective therapeutic impact on deep Achilles tendon injuries, as verified through pathological assessments and biomechanical analysis (Figures 3, 4). Thus, the novel high-power LTP device has the potential to treat deep tissue and organ diseases, significantly expanding the range of plasma applications in clinical treatment.

Collagen, a triple-helical protein primarily involved in mechanical activity, is crucial for providing tissues with tensile strength and is essential for tissue repair (Prabhu et al., 2014). It is widely recognized that during the acute phase of wound healing, levels of type III collagen increase, leading to the formation of scar tissue. Subsequently, as the healing process progresses, type I collagen replaces type III collagen. Injured tendons undergo a similar process (Oshita et al., 2016; Bailey et al., 1975). The optimal ratio of type III to type I collagen may significantly influence the quality of tendon healing (Cui et al., 2023). After repeated or overuse repeated or overuse injuries, tendons may retain an aberrant collagen ratio, characterized by a high ratio of type III collagen to type I collagen. Persistent abnormal collagen ratios can weaken tendons over time, increasing the risk of mechanical stress and degenerative changes in the tendons (Oshita et al., 2016). Previous research suggests that LTP treatment enhances the expression of type I collagen in skin fibroblasts, thereby promoting wound healing (Arndt et al., 2013). Additionally, LTP treatment has been shown to promote the expression of type I collagen in wound tissue and accelerate wound closure significantly (Cui et al., 2020). In the current study, the expression of type III collagen, which is essential for the acute phase of tendon repair, was significantly higher in both the model and LTP groups compared to the control group. Interestingly, the expression of type I collagen was significantly higher, and the ratio of type III collagen to type I collagen was significantly lower in the LTP group compared to the model group (Figure 5). This suggests that LTP treatment facilitates the transformation of collagen, thereby normalizing collagen ratios and improving tendon structure, which ultimately enhances tendon strength. Consistently, the LTP group demonstrated significantly better performance than the model group in four biomechanical indices: maximum stress, maximum load, stiffness, and Young’s modulus (Figure 3). Therefore, the high-power LTP device utilized in this study has the potential to promote the synthesis of type I collagen within tendon tissues, thereby aiding in the healing of tendon disorders.

Proinflammatory cytokines such as IL-1β are recognized as primary initiators of tendinopathy and can be released by tenocytes in response to mechanical overload (Wu et al., 2019). Studies have demonstrated that IL-1β can significantly suppress the expression of collagen type I and other extracellular matrix compounds specific to tendons in tenocytes, leading to tendon degradation and loss of biomechanical structural integrity (Shakibaei et al., 2011). Furthermore, the overexpression of inflammatory mediators like TNF-α and IL-1β promotes the synthesis of matrix metalloproteinases (MMPs) (Burrage et al., 2006). MMPs are known to play a role in tendon matrix degradation, predisposing individuals to painful tendinopathy and tendon rupture (Baugé et al., 2015; Del Buono et al., 2013). Therefore, the inhibition of inflammation is crucial in protecting tendons from pathological processes. Recent research has explored the use of biomaterials and anti-inflammatory medications for the treatment of tendinopathy. For example, Hyaluronic acid nanofibrous membranes filled with ibuprofen have been shown to reduce inflammation and prevent tendon adhesion following surgery. (Chen et al., 2019). Additionally, fullerenol has been found to inhibit tendinopathy by alleviating inflammation (Jiao et al., 2023). Consistently, our findings demonstrated that the expression of matrix metalloproteinases MMP-1 and MMP-2, as well as inflammatory markers IL-1β, TNF-α, and COX-2, was significantly reduced by LTP therapy (as depicted in Figure 6). This suggests that LTP therapy may effectively treat tendinopathy by inhibiting inflammation and subsequently reducing matrix metalloproteinase expression.

Nitric oxide (NO), a small free radical generated by the nitric oxide synthase (NOS) family, plays a significant role in regulating the tendon healing process. NO is induced by all three isoforms of NOS, and NOS activity is upregulated in tendinopathy (Szomor et al., 2006; Bokhari and Murrell, 2012). In a rat Achilles tendon injury model, the NOS inhibitor L-NAME has been shown to inhibit tendon healing and collagen synthesis (Tomiosso et al., 2009). Conversely, additional NO has been found to enhance tendon healing by increasing collagen content, promoting better collagen reorganization, and elevating ultimate stress (Murrell et al., 2008; Yuan et al., 2003). Moreover, clinical trials have demonstrated that delivering NO enhances the recovery of conditions such as Achilles tendonitis, chronic lateral epicondylitis, and supraspinatus tendinopathy. These improvements are accompanied by alleviated clinical symptoms, including a reduction in pain, an increase in range of motion, and enhanced strength (Bokhari and Murrell, 2012). In-depth studies have revealed several potential molecular mechanisms underlying these effects. Firstly, NO produced by inducible nitric oxide synthase (iNOS) can induce apoptosis in inflammatory cells, thereby preventing the growth of chronic inflammation by eliminating inflammatory cells and reorganizing inflammatory areas (Akiba et al., 1998; Lin et al., 2001). Secondly, NO exerts a vasodilatory function, leading to an increase in local blood flow (Sessa, 2009). Thirdly, NO contributes to the accumulation of tendon collagen and enhances the acquisition of mechanical strength (Xia et al., 2006). In the present study, NO is a chemically active substance richly produced by the novel high-power LTP device. It can effectively and percutaneously penetrate into Achilles tendon tissue and blood (Figures Figure7A,B). Furthermore, consistent with these findings, molecular examination revealed inhibited inflammatory responses (Figure 6), promoted expression of type I collagen (Figure 5), and facilitated blood flow (Figures 7C,D) in injured Achilles tendons following LTP treatment. These evidences suggest that NO produced by LTP may play a crucial role in promoting the healing of tendon injuries.

This study has several limitations to note. First, the current data primarily clarify the correlation between LTP and tendon tissue responses - including collagen conversion, inflammation, and blood perfusion - mediated by NO. This provides a foundation for exploring tendinopathy therapeutic mechanisms. However, fully elucidating LTP’s therapeutic principles requires subsequent causality verification, as both correlation analysis and causality confirmation are essential. Second, while we demonstrated LTP’s therapeutic effects on tendinopathy, its underlying molecular mechanisms remain unclear and need further investigation. Third, our research focused solely on NO (an active LTP component) in treating injured tendons, leaving other active components’ roles unexplored.

## 5 Conclusion

In conclusion, our findings indicate that LTP plays a pivotal role in attenuating inflammation, facilitating collagen transformation, and enhancing blood flow, thereby improving the biomechanical properties of the injured tendon and supporting its healing process. Moreover, NO was identified as playing a significant role in these processes. These results unveil new therapeutic prospects and provide clinical evidence of LTP’s efficacy in treating tendinopathy and tendon injuries, while also yielding valuable insights into the potential of LTP in managing deep tissue and organ diseases.

## Data Availability

The original contributions presented in the study are included in the article/supplementary material, further inquiries can be directed to the corresponding authors.

## Abbreviations

BSA, bovine serum albumin; COX-2, cyclooxygenase-2; DAB, diaminobenzidine; IL-1β, interleukin 1-β; LTP, Low-temperature plasma; MMP-1, matrix metalloproteinase1; MMP-2, matrix metalloproteinase2; NO, nitric oxide; OES, optical emission spectroscopy; RT-qPCR, reverse transcription-polymerase chain reaction; TNF-α, tumor necrosis factor alpha.
